# Assessment of Artemisinin Contents in Selected *Artemisia* Species from Tajikistan (Central Asia)

**DOI:** 10.3390/medicines6010023

**Published:** 2019-01-31

**Authors:** Sodik Numonov, Farukh Sharopov, Aminjon Salimov, Parviz Sukhrobov, Sunbula Atolikshoeva, Ramazon Safarzoda, Maidina Habasi, Haji Akber Aisa

**Affiliations:** 1 Research Institution “Chinese-Tajik Innovation Center for Natural Products” of the Tajikistan Academy of Sciences, Ayni str. 299/2, Dushanbe 734063, Tajikistan; sodikjon82@gmail.com (S.N.); shfarukh@mail.ru (F.S.); 2 Key Laboratory of Plant Resources and Chemistry in Arid Regions, Xinjiang Technical Institute of Physics and Chemistry, Chinese Academy of Sciences, Urumqi 830011, China; parviz@gmail.com (P.S.); sunbula87@mail.ru (S.A.); 3 Center for Research in Innovative Technologies, Academy of Sciences of the Republic of Tajikistan, Dushanbe 734062, Tajikistan; 4 Department of Pharmaceutical Technology, Avicenna Tajik State Medical University, Rudaki 139, Dushanbe 734003, Tajikistan; safarzoda90@yandex.ru; 5 V.I. Nikitin Institute of Chemistry of the Tajikistan Academy of Sciences, Ayni str. 299/2, Dushanbe 734063, Tajikistan; amin-jon-86@mail.ru

**Keywords:** *Artemisia* species, *Artemisia vachanica*, artemisinin, HPLC-PAD, Tajikistan

## Abstract

**Background:** Central Asia is the center of origin and diversification of the *Artemisia* genus. The genus *Artemisia* is known to possess a rich phytochemical diversity. Artemisinin is the shining example of a phytochemical isolated from *Artemisia annua*, which is widely used in the treatment of malaria. There is great interest in the discovery of alternative sources of artemisinin in other *Artemisia* species. **Methods:** The hexane extracts of *Artemisia* plants were prepared with ultrasound-assisted extraction procedures. Silica gel was used as an adsorbent for the purification of *Artemisia annua* extract. High-performance liquid chromatography with ultraviolet detection was performed for the quantification of underivatized artemisinin from hexane extracts of plants. **Results:** Artemisinin was found in seven *Artemisia* species collected from Tajikistan. Content of artemisinin ranged between 0.07% and 0.45% based on dry mass of *Artemisia* species samples. **Conclusions:** The artemisinin contents were observed in seven *Artemisia* species. *A. vachanica* was found to be a novel plant source of artemisinin. Purification of *A. annua* hexane extract using silica gel as adsorbent resulted in enrichment of artemisinin.

## 1. Introduction

As reported by the World Health Organization (WHO), in the last four years (2015–2018), nearly half of the population of the world (3.2 billion people) was at risk of malaria. In 2017, there were 435,000 deaths from malaria globally, 61% (266,000) of which were of children younger than five years [1]. 

At present, artemisinin-based combination treatments are effective and accepted as being among the best malaria treatments [2]. Artemisinin is the bioactive compound produced by the plant *Artemisia annua* L. Artemisinin has saved the lives of millions of malarial patients worldwide and served as the standard regimen for treating *Plasmodium falciparum* infection [3].

With 500 species, the genus *Artemisia* L. is the largest and the most widely distributed genus of the Asteraceae, and Central Asia is the center of origin and diversification of the genus [4]. Many *Artemisia* species grow in Tajikistan [5]. The genus *Artemisia* is known to possess rich phytochemical diversity [6,7,8,9]. Almost 600 secondary metabolites have been characterized from *A. annua* alone [10]. 

**Figure 1:**
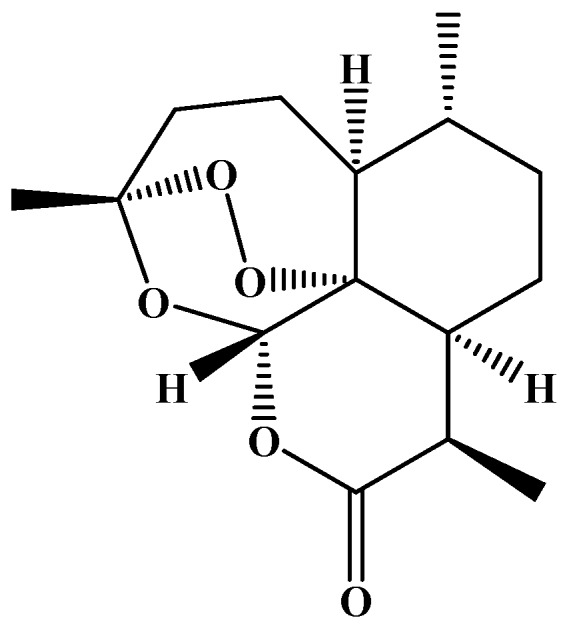
Artemisinin structure.

Artemisinin is the shining example of a phytochemical isolated from *A. annua*, and is widely used in the treatment of malaria. Artemisinin is a natural sesquiterpene lactone with an unusual 1,2,4-trioxane substructure (Figure 1). It is soluble in most aprotic solvents and is poorly soluble in water. It decomposes in protic solvents, probably by the opening of the lactone ring [11]. The artemisinin biosynthesis proceeds via the tertiary allylic hydroperoxide, which is derived from the oxidation of dihydroartemisinic acid [10].

The mechanism of artemisinin action is controversial [12,13]. It is related to the presence of an endoperoxide bridge, which by breaking creates a powerful free radical form of the artemisinin, which attacks the parasite proteins without harming the host [14].

The presence of artemisinin has been reported in many *Artemisia* species, including *A. absinthium*, *A. anethifolia*, *A. anethoides*, *A. austriaca*, *A. aff. tangutica*, *A. annua*, *A. apiacea*, *A. bushriences*, *A. campestris*, *A. cina*, *A. ciniformis*, *A. deserti*, *A. diffusa*, *A. dracunculus*, *A. dubia*, *A. incana*, *A. indica*, *A. fragrans*, *A. frigida*, *A. gmelinii*, *A. japonica*, *A. khorassanica*, *A. kopetdaghensis*, *A. integrifolia*, *A. lancea*, *A. macrocephala*, *A. marschalliana*, *A. messerschmidtiana*, *A. moorcroftiana*, *A. parviflora*, *A. pallens,* *A. roxburghiana*, *A. scoparia*, *A. sieberi*, *A. sieversiana*, *A. spicigeria*, *A. thuscula*, *A. tridentata*, *A. vestita*, and *A. vulgaris* [9,15,16,17,18,19,20,21,22,23,24,25,26,27,28,29,30,31,32]. 

The chemical structure of artemisinin provided a foundation for several synthetic antimalarial drugs including pyronaridine, lumefantrine (benflumetol), naphthoquine, and so on [33]. Recently, research interest in biotechnological approaches for enhanced artemisinin production in *Artemisia* have increased due to the global needs and low amounts of artemisinin and its derivatives in *Artemisia* plants [34]. Various biotechnological approaches such as the transformation of genes for production of artemisinin to cells of eukaryotic and prokaryotic organisms and to genetically engineered yeast were developed to enhance the production of artemisinin and its derivatives [35,36,37,38]. 

In addition, artemisinin and its bioactive derivatives have a second career as antitumor agents [39]. They demonstrated high efficiency against a variety of cancer cells, with minor side effects to normal cells in cancer patients [40,41]. The investigation of the biological activity of *Artemisia* species and their constituents is required to explore the full potential of diverse *Artemisia* species and their chemical ingredients against cancer, malaria, and infections [42]. Artemisinin can also exert beneficial effects in treatment of the wide-spectrum diseases such as obesity, diabetes, and aging-related disorders [43]. 

Natural conditions influence the biotransformation and accumulation of artemisinin in plants. For example, Ferreira et al. reported that that biosynthesis of artemisinin is affected by light intensity [44]. The relatively large number of sunny days per year in Tajikistan is essential for artemisinin accumulation in *Artemisia* species.

Accordingly, there is great interest in the discovery of artemisinin in *Artemisia* species growing wild in Tajikistan. The purpose of the current investigation was to evaluate the presence of artemisinin in eight *Artemisia* species.

## 2. Materials and Methods

### 2.1. Plant Materials

The aerial parts of eight *Artemisia* species including *A. annua, A. vachanica, A. vulgaris, A. makrocephala, A. leucotricha, A. dracunculus, A. absinthium*, and *A. scoparia* were collected during their vegetative and flowering period from three regions of Tajikistan. The voucher specimen numbers, local names, collection time, and location of plants are summarized in Table 1. These species were identified with regards to specimens in the herbarium of the Institute of Botany, Plant Physiology and Genetics of Tajikistan Academy of Sciences. The voucher specimens of the plant material were deposited at the Chinese‒Tajik Innovation Center for Natural Products research institution of the Tajikistan Academy of Sciences.

### 2.2. Preparation of Hexane Extracts

The extraction process of dried aerial parts of *Artemisia* plants were prepared by the following procedure: 10 g of plant materials were crushed into smaller pieces and weighed in a 250-mL flask, into which 150 mL of hexane were added at room temperature. The prepared plant mixtures were sonicated in an ultrasonic bath at a frequency of 35 kHz for 15 min at room temperature. Then, plant mixtures were allowed to stand for 12 h at room temperature. After 12 h, they were filtered through Whatman filter paper and used for the designed chemical analysis.

The yield of hexane extracts were calculated using following Equation (1):

(1) $$\omega \left(\%\right)=\frac{a*100\%}{b},$$

where ω is the yield of hexane extract (%), *a* is the weight of hexane extract; and *b* is the weight of plant sample. 

### 2.3. Quantitative Analysis of Artemisinin Using HPLC

A number of studies have been addressed for the development of HPLC methodology for quantification of artemisinin in plant material and extracts [44,45]. The best separation of artemisinin was achieved on columns Luna 5 μm C18 250 × 4.6 mm (Phenomenex, Torrance, CA, USA) and Betasil C18 5 μm 250 × 4.6 mm (Thermo Fisher Scientific, Waltham, MA, USA), using acetonitrile:water (65:35, *v*/*v*) as the mobile phase [44,45]. 

In addition, artemisinin analysis by HPLC-PAD at 192 nm, compared to HPLC with evaporative light scattering detection (HPLC-ELSD), was very accurate, precise, and reproducible [44]. 

*Artemisia* extracts were analyzed by HPLC UltiMate 3000 system with DAD detector (Thermo Fisher Scientific, Waltham, MA, USA). Extracts of *Artemisia* plants (10 mg/mL) were prepared in methanol and the solution was filtered using a 0.45-µm syringe filter for HPLC analysis. Analysis was performed on a Waters Bridge C18 5 μm (250 × 4.6 mm, Waters, Milford, MA, USA) and XSelect CSH^TM^ C18 5 μm (250 × 4.6 mm, Waters, Milford, MA, USA) columns. The mobile phase consisted of water (A) and acetonitrile (B). The gradient elution program was as follows: 0–7 min, hold 60% of B; 17–30 min, 60–100% of B; 30–35 min, 100% of B. The detection wavelengths were 192, 210, 254, and 320 nm, the flow rate was 1 mL/min, the injection volume was 5 µL, and the oven temperature was set to 30 °C.

**Figure 2:**
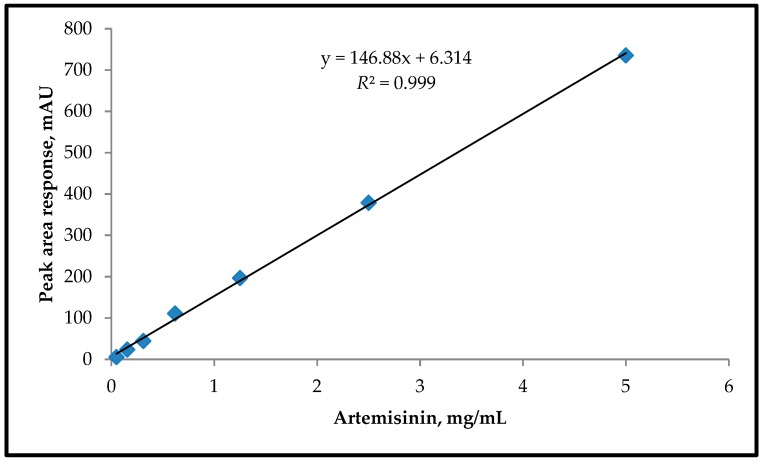
Linear calibration graph for the standard artemisinin.

Quantification of the artemisinin was performed using a linear calibration graph with increasing amounts of artemisinin and their peak area response with UV detection (192 nm) (Figure 2). Standard solutions with seven different concentrations (between 0.05 and 5 mg/mL) were prepared by solving the standard artemisinin in methanol.

### 2.4. Purification of Artemisia annua Extract

Silica gel as adsorbent (10.0 g dry weight) was added to 100 mL of *A. annua* extract (10 mg/mL in hexane) in a 250-mL flask while agitating on a shaker at room temperature until the adsorption reached equilibrium. After reaching the adsorption equilibrium, the silica gel was filtered from the mixture and then washed with hexane 3–4 times until decolorization of the filtrate. The filtrate was evaporated using a rotary evaporator.

## 3. Results

The yields of hexane extract, number of components detected at 192 nm, and content of artemisinin per dry weight plant are summarized in Table 2. The hexane percentage yield of *Artemisia* species ranged from 2.3% to 8.1%. The hexane extract of *A. vachanica* had the highest yield (8.1%), followed by *A. annua* (5.8%) and *A. absinthium* (5.4%), while *A. vulgaris* had the lowest yield (2.3%). A total of 83, 90, 95, 94, 75, 100, 98, and 90 components (peaks on the chromatograms) were detected in *A. annua*; *A. vachanica*; *A. vulgaris*; *A. makrocephala*; *A. leucotricha*; *A. dracunculus*; *A. absinthium*, and *A. scoparia,* respectively. 

The content of artemisinin per dry weight of *Artemisia* species ranged from 0.07% to 0.45%. The highest content of artemisinin was observed in *A. annua* (0.45%), followed by *A. vachanica* (0.34%), while *A. dracunculus* had the lowest artemisinin content (0.07%). The HPLC chromatograms of *Artemisia* species with standard of artemisinin are showed in Figures S1–S5. 

**Figure 3:**
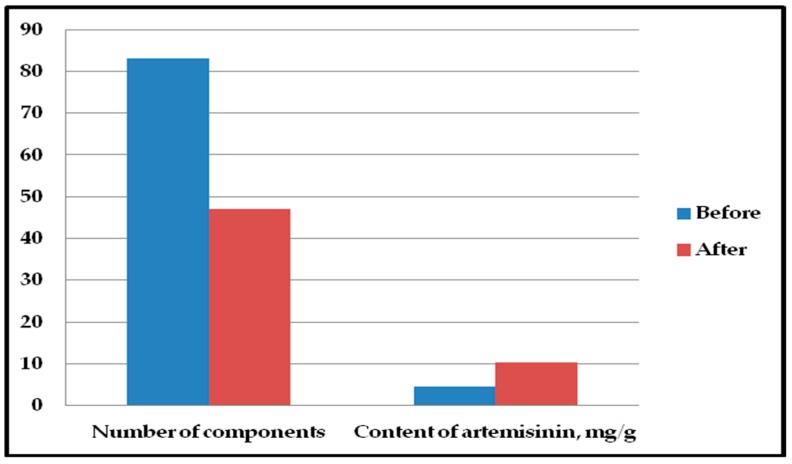
Total components number and artemisinin content in *A. annua* extract before and after purification.

After treatment of *A. annua* extract with silica gel as an adsorbent, the total peaks in the chromatograms decreased from 83 to 47, while the content of artemisinin in *A. annua* extract increased from 4.5 mg/g to 10.2 mg/g. The results of *A. annua* extract treatment are given in Figure 3.

## 4. Discussion

There are many factors such as environmental, genetic, etc. that can influence the variation in artemisinin concentration [17]. Ranjbar and co-authors have reported that there is a relationship between the increased expression of some genes and the enhancement of artemisinin content in *Artemisia* species at the vegetative, budding, and flowering stages [22]. Recently, Salehi et al. investigated the expression of artemisinin biosynthesis and trichome formation genes in five *Artemisia* species and concluded that there is a relationship between the enhancement of artemisinin content and increased expression of some genes [23].

Previous works have reported that artemisinin concentration varied due to differences in methods of artemisinin extraction as well as the solvents used [40,41]. Literature reports indicated that extraction of artemisinin has been carried out by different extraction methods: traditional solvent extraction, microwave-assisted extraction, ultrasound-aided extraction, and supercritical fluid extraction method using CO_2_ as a solvent [20,46]. *n*-hexane [38], toluene [40], chloroform [41], petroleum ether [44], acetone, and ethanol [47] were the solvents most widely used for artemisinin extraction from *Artemisia* species. 

According to our results, ultrasound-aided extraction and *n*-hexane as a solvent for artemisinin extraction were suitable for the extraction of artemisinin from *Artemisia* species. Our experiments are in agreement with previous reports that the yield of artemisinin extraction is enhanced by ultrasound-aided extraction when compared to comparable conventional extraction processes [46].

Various methods such as thin-layer chromatography (TLC), high-performance liquid chromatography (HPLC) with evaporative light scattering detection (ELSD), ultraviolet detection (PAD), diode array detection (DAD), gas chromatography (GC) combined with flame ionization detector (FID), and mass spectrometry (MS) have been proposed and assessed to detect and quantify artemisinin [48,49]. Recently, a fully electrochemical molecularly imprinted polymer sensor was developed for the sensitive detection of artemisinin with a detection limit of 0.02 μM in plant matrix [50].

In the present work, HPLC-PAD was used to analyze artemisinin in *Artemisia* species. Researchers have reported that HPLC-PAD is readily applicable for quality control of herbals and artemisinin-related pharmaceutical compounds and it was validated for the quantification of underivatized artemisinin, dihydroartemisinic acid, and artemisinic acid from crude plant samples [44]. 

The literature reports with respect to artemisinin content in *Artemisia* species up to now are summarized in Table 3. Artemisinin was found at least in 40 *Artemisia* species [9,15,16,17,18,19,20,21,22,23,24,25,26,27,28,29,30,31,32]. The artemisinin content ranged from 0.0005% to 1.38% on dried parts of *Artemisia* species. 

The highest content of artemisinin was found in *A. annua* (up to 1.4%) [51], followed by *A. deserti* (0.4–0.6%), *A. marschalliana* (up to 0.38%) [23] and *A. absinthium* (up to 0.35%) [17,22,24]. 

The current study observed the presence of artemisinin in eight of the various *Artemisia* species growing wild in Tajikistan. *A. vachanica* was found to be a novel plant source of artemisinin. The content of artemisinin in *A. macrocephala* was found almost 20-fold higher than a previous study [30]. Hence, no significant difference was detectable between the artemisinin content of *A. annua*, *A. absinthium*, *A. dracunculus*, and *A. vulgaris* growing in Tajikistan. Generally, previous studies reported that the artemisinin content in *A. annua* was higher than in other *Artemisia* species [15,17,21,22,24]. Artemisinin was not detected in *A. leucotricha*. 

The extracts obtained from plant material using organic solvent extractions are very complex, and have several unwanted components such as chlorophylls and other colored organic molecules from the feed material. Removal of the contaminants from the extracts has been performed with charcoal and clays [53,54]. 

In this work, purification of *A. annua* hexane extract by using silica gel as adsorbent resulted in the enrichment of artemisinin. The concentration of artemisinin increased 2.3 times and 35 unwanted components were purified out by silica gel. 

A selective sorption method has resulted in the increase of artemisinin to a final purity up to 90% using a polymeric adsorbent loaded with specific ligands [53]. Using silica gel compared to an adsorbent with specific ligands was less effective. However, silica gel is a cheap alternative that can be used for primary treatment of crude artemisinin extracts from the feed material. 

## 5. Conclusions

The study demonstrated the presence of artemisinin, a biologically important natural sesquiterpene lactone in several *Artemisia* species growing wild in Tajikistan. The content of artemisinin ranged between 0.07% and 0.45% on a dry weight basis of *Artemisia* species. *A. vachanica* was found to be a novel plant source of artemisinin. Treatment of *A. annua* hexane extract with silica gel as adsorbent resulted in enrichment of artemisinin. 

## Figures and Tables

**Table 1:** *Artemisia* species collected from Tajikistan.

Species	Local Name	Collection Site	Time	Voucher Number
*A. annua*	гoвҷoрӯб (govjorub), бурғун (burghun)	Ziddeh, Varzob Region	15.07.2018	CTICNPG 2018 - 5
*A. vachanica*	пуши oддӣ (pushi oddi)	Khaskhorugh, Ishkoshim Region	25.08.2018	CTICNPG 2018 - 6
*A. vulgaris*	cафедҷoрӯб (safedjorub), явшoн (yavshon)	Khaskhorugh, Ishkoshim Region	22.08.2018	CTICNPG 2018 - 7
*A. mackrocephala*	пуши калoнгул (pushi kalongul)	Khaskhorugh, Ishkoshim Region	21.08.2018	CTICNPG 2018 - 8
*A. leucotricha*	пуши сафед (pushi safed)	Khaskhorugh, Ishkoshim Region	22.08.2018	CTICNPG 2018 - 9
*A. dracunculus*	тархун (tarkhun), ғӯда (ghuda)	Ziddeh, Varzob Region	15.07.2018	CTICNPG 2018 - 10
*A. absinthium*	тaхач (takhach)	Guli Bodom, Yovon Region	20.07.2018	CTICNPG 2018 - 11
*A. scoparia*	туғак (tughak), маҳинҷoрӯб (mahinjorub)	Guli Bodom, Yovon Region	20.07.2018	CTICNPG 2018 - 12

**Table 2:** Extraction yield, componential composition, and artemisinin content in *Artemisia* species.

Species	Yield of Hexane Extract, %	Number of Components	Content of Artemisinin in Dry Weight Plant, %
*Artemisia annua*	5.80 ± 0.05	83	0.45 ± 0.03
*Artemisia vachanica*	8.09 ± 0.1	90	0.34 ± 0.02
*Artemisia vulgaris*	2.32 ± 0.02	95	0.18 ± 0.01
*Artemisia makrocephala*	3.01 ± 0.02	94	0.20 ± 0.01
*Artemisia leucotricha*	3.19 ± 0.03	75	Not detected
*Artemisia dracunculus*	3.78 ± 0.04	100	0.07 ± 0.01
*Artemisia absinthium*	5.41 ± 0.0.5	98	0.09 ± 0.01
*Artemisia scoparia*	3.39 ± 0.02	90	0.11 ± 0.02

**Table 3:** Summary of literature reports showing presence of artemisinin in *Artemisia* species.

Artemisia Species	Part Used	Artemisinin, %	Ref.
*A. absinthium*	Flowers, leaves, stem, and roots	0.02–0.35	[17,22,24]
*A. austriaca*	Leaves	0.05	[23]
*A. aff. tangutica*	Flowers, leaves, stem, and roots	0.02–0.11	[17]
*A. anethifolia*	Leaves	0.05	[30]
*A. anethoides*	Leaves	0.006	[30]
*A. annua*	Flowers, leaves, stem, and roots	0.02–1.4	[17,25,26,27,28,51,52]
*A. arborescens*	Leaves	0.001	[30]
*A. apiacea*	Leaves	Not shown	[21]
*A. bushriences*	Flowers, leaves, stem, and roots	0.01–0.34	[17]
*A. campestris*	Leaves, buds, and flowers	0.05–0.1	[22]
*A. cina*	Shoots	0.0006	[16]
*A. ciniformis*	Leaves	0.22	[23]
*A. deserti*	Leaves	0.4–0.6	[23]
*A. diffusa*	Leaves, buds, and flowers	0.05–0.15	[22]
*A. dracunculus*	Flowers, leaves, stem, and roots	0.01–0.27	[17]
*A. dubia*	Flowers, leaves, stem, and roots	0.01–0.07	[17,19]
*A. incana*	Leaves	0.25	[23]
*A. indica*	Flowers, leaves, stem, and roots	0.01–0.10	[17,19]
*A. fragrans*	Leaves	0.2	[23]
*A. frigida*	Leaves	0.007	[30]
*A. gmelinii*	Leaves	0.038	[30]
*A. japonica*	Flowers, leaves, stem, and roots	0.01–0.08	[17]
*A. kopetdaghensis*	Leaves	0.18	[23]
*A. integrifolia*	Leaves	0.036	[30]
*A. lancea*	Leaves	Not shown	[21]
*A. macrocephala*	Leaves	0.011	[30]
*A. marschalliana*	Leaves	0.38	[23]
*A. messerschmidtiana*	Leaves	0.032	[30]
*A. moorcroftiana*	Flowers, leaves, stem, and roots	0.01–0.16	[17]
*A. parviflora*	Flowers, leaves, stem, and roots	0.03–0.15	[17]
*A. pallens*	Leaves and flowers	0.1	[31]
*A. roxburghiana*	Flowers, leaves, stem, and roots	0.02–0.22	[17]
*A. scoparia*	Leaves, buds, and flowers	0.1–0.18	[22,32]
*A. sieberi*	Aerial parts	0.1–0.2	[22,23]
*A. sieversiana*	Flowers, leaves, stem, and roots	0.05–0.20	[17]
*A. spicigeria*	Leaves, buds, and flowers	0.05–0.14	[22]
*A. thuscula* (syn. *A. canariensis*)	Leaves	0.045	[30]
*A. tridentata* Nutt. subsp. *vaseyana*	Leaves	0.0005	[30]
*A. vestita*	Flowers, leaves, stem, and roots	0.04–0.20	[17]
*A. vulgaris*	Flowers, leaves, stem, and roots	0.02–0.18	[17,22]

## Supplementary Materials

The following are available online at http://www.mdpi.com/2305-6320/6/1/23/s1, Figure S1: HPLC chromatogram of the pure artemisinin (Rt 4.257 min) (A) and hexane extract of *Artemisia annua* (B), Figure S2: HPLC chromatogram of hexane extract of *Artemisia vachanica* (A) and *Artemisia vulgaris* (B), Figure S3: HPLC chromatogram of hexane extract of *Artemisia makrocephala* (A) and *Artemisia leucotricha* (B), Figure S4: HPLC chromatogram of hexane extract of *Artemisia dracunculus* (A) and *Artemisia absinthium* (B), Figure S5: HPLC chromatogram of hexane extract of *Artemisia scoparia* (A) and mixture *Artemisia annua* and pure artemisinin (B).
