# Supplementary material for: Assessment of Artemisinin Contents in Selected Artemisia Species from Tajikistan (Central Asia)
Source: Medicines (Basel). 2019 Jan 31;6(1):23. doi: 10.3390/medicines6010023 (PMC6473495; doi:10.3390/medicines6010023)
Supplement: Supplementary file 1 [file medicines-06-00023-s001.pdf]

Supplementary Materials: Assessment of Artemisinin Contents in Selected *Artemisia* Species from Tajikistan (Central Asia)

Sodik Numonov, Farukh Sharopov, Aminjon Salimov, Parviz Sukhrobov, Sunbula Atolikshoeva, Ramazon Safarzoda, Maidina Habasi and Haji Akber Aisa

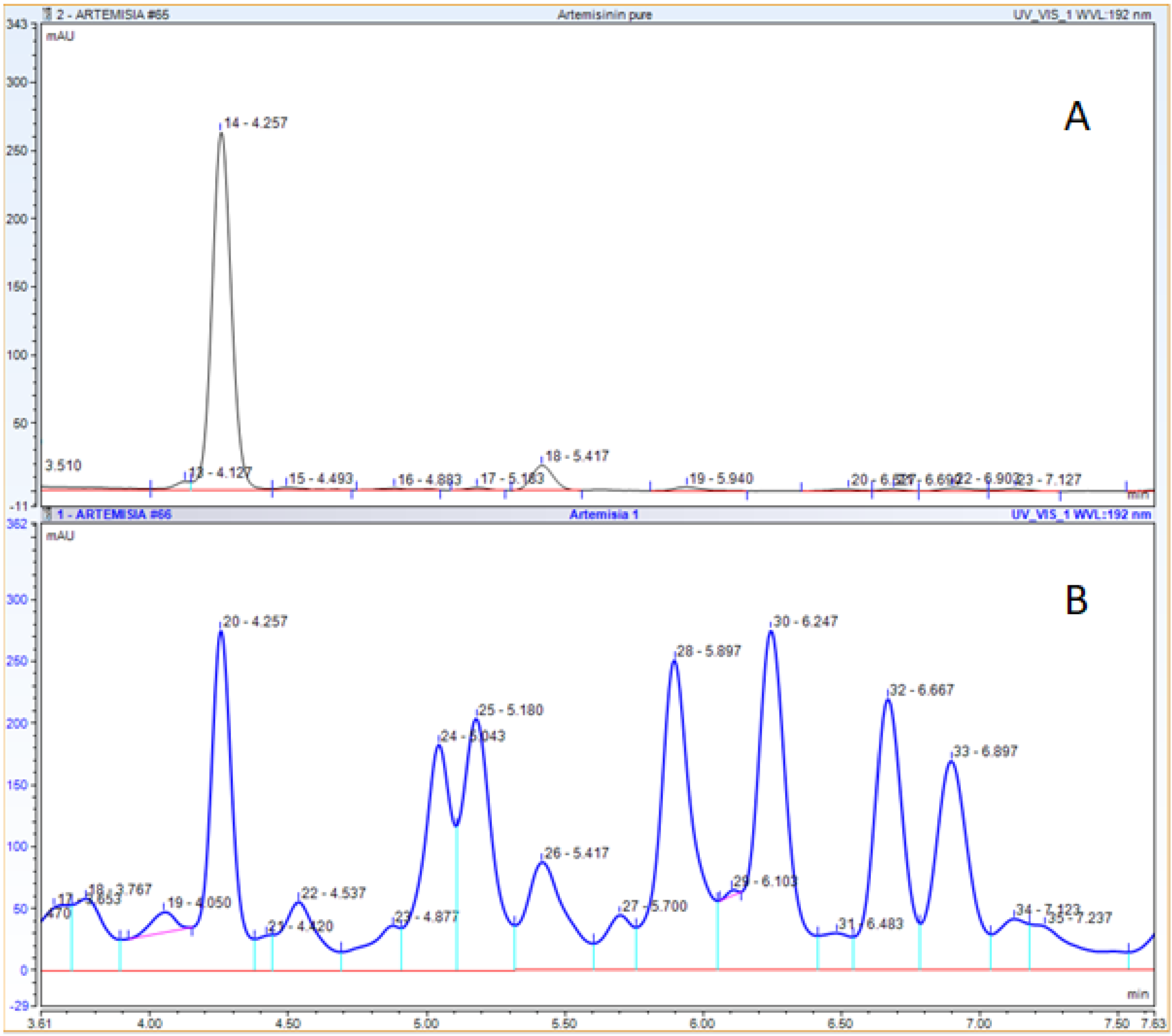

Figure S1. HPLC chromatogram of the pure artemisinin (Rt 4.257 min) (**A**) and hexane extract of *Artemisia annua* (**B**).

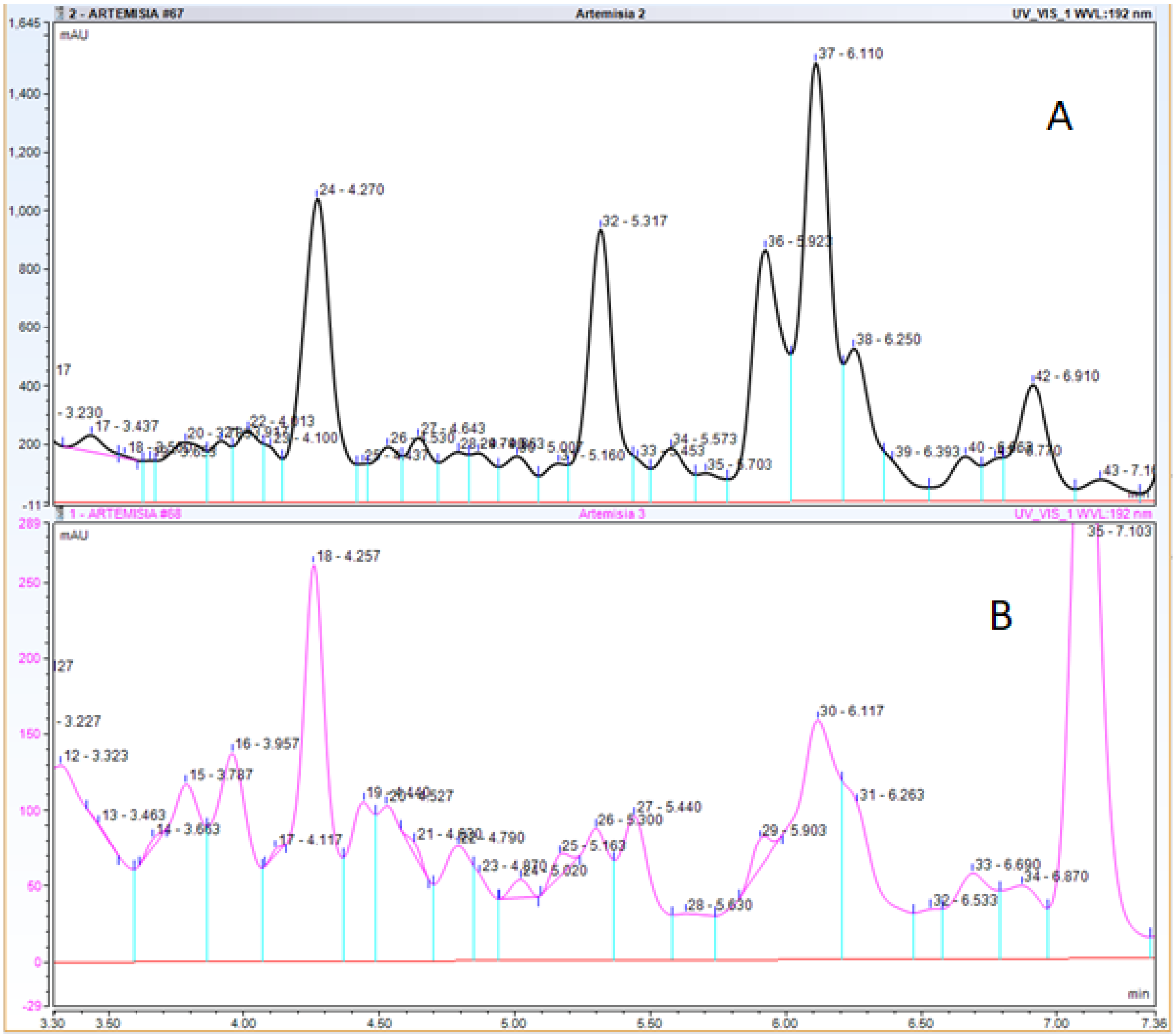

Figure S2. HPLC chromatogram of hexane extract of *Artemisia vachanica* (**A**) and *Artemisia vulgaris* (**B**).

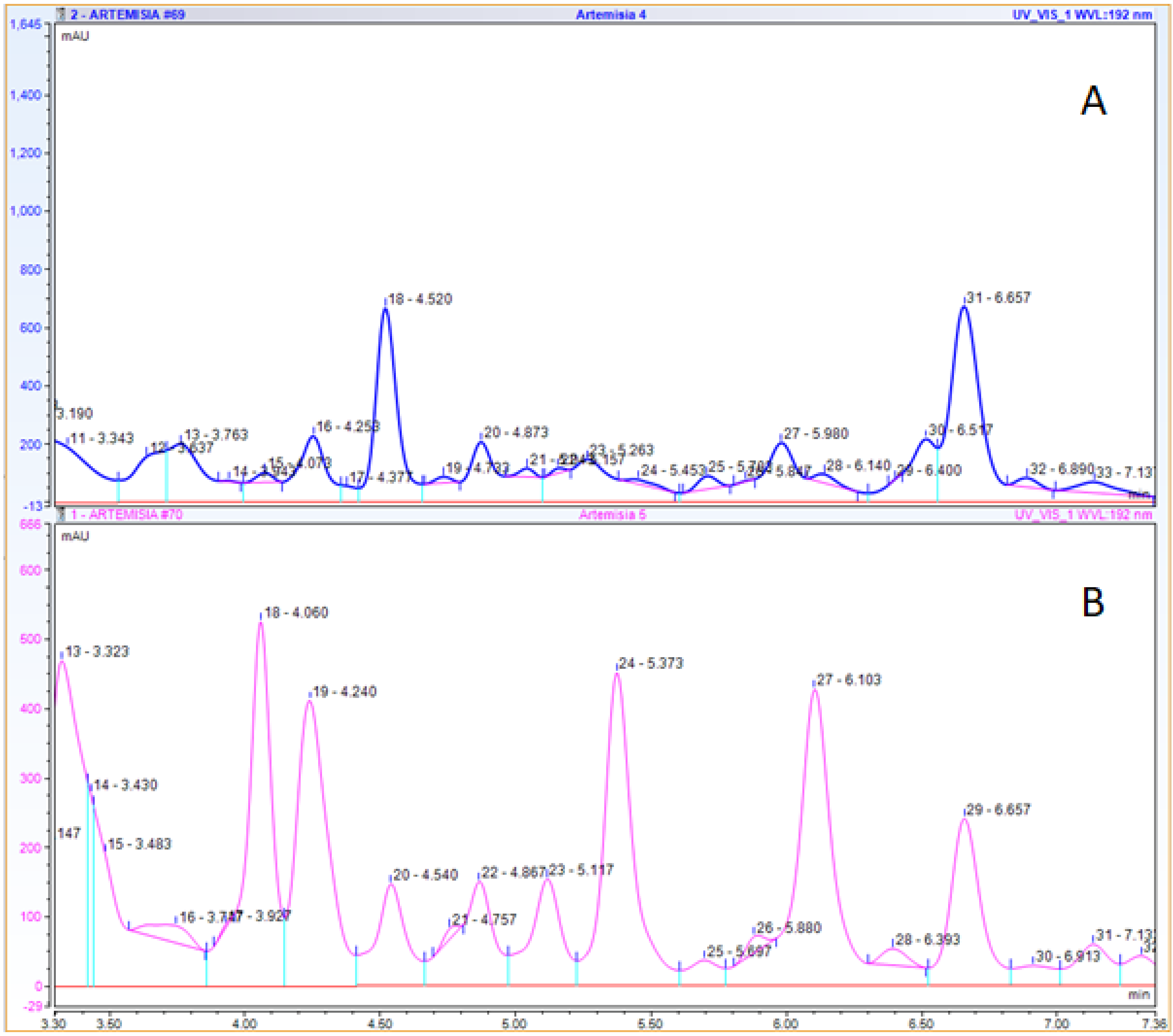

Figure S3. HPLC chromatogram of hexane extract of *Artemisia makrocephala* (**A**) and *Artemisia leucotricha* (**B**).

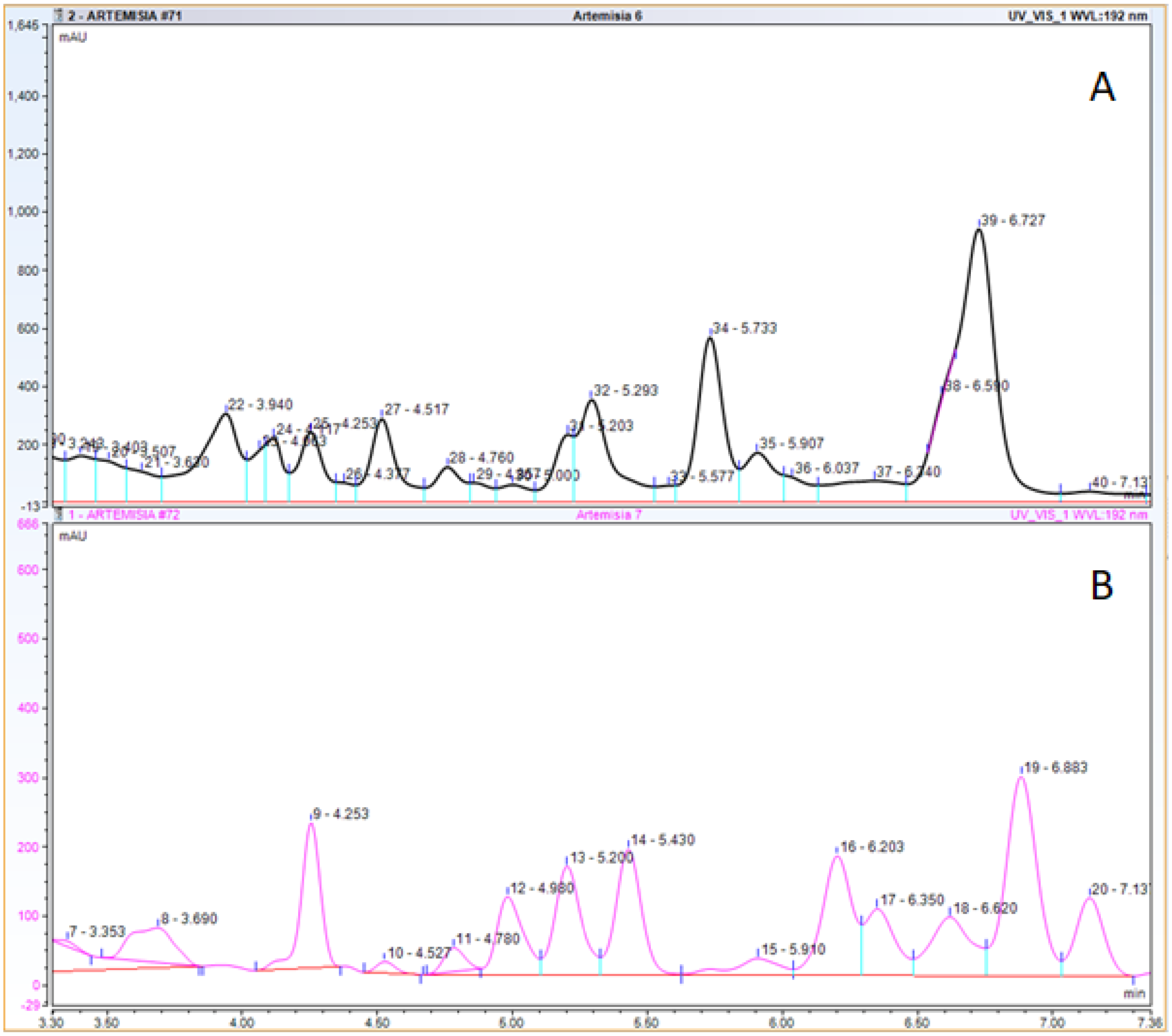

Figure S4. HPLC chromatogram of hexane extract of *Artemisia dracunculus* (**A**) and *Artemisia absinthium* (**B**).

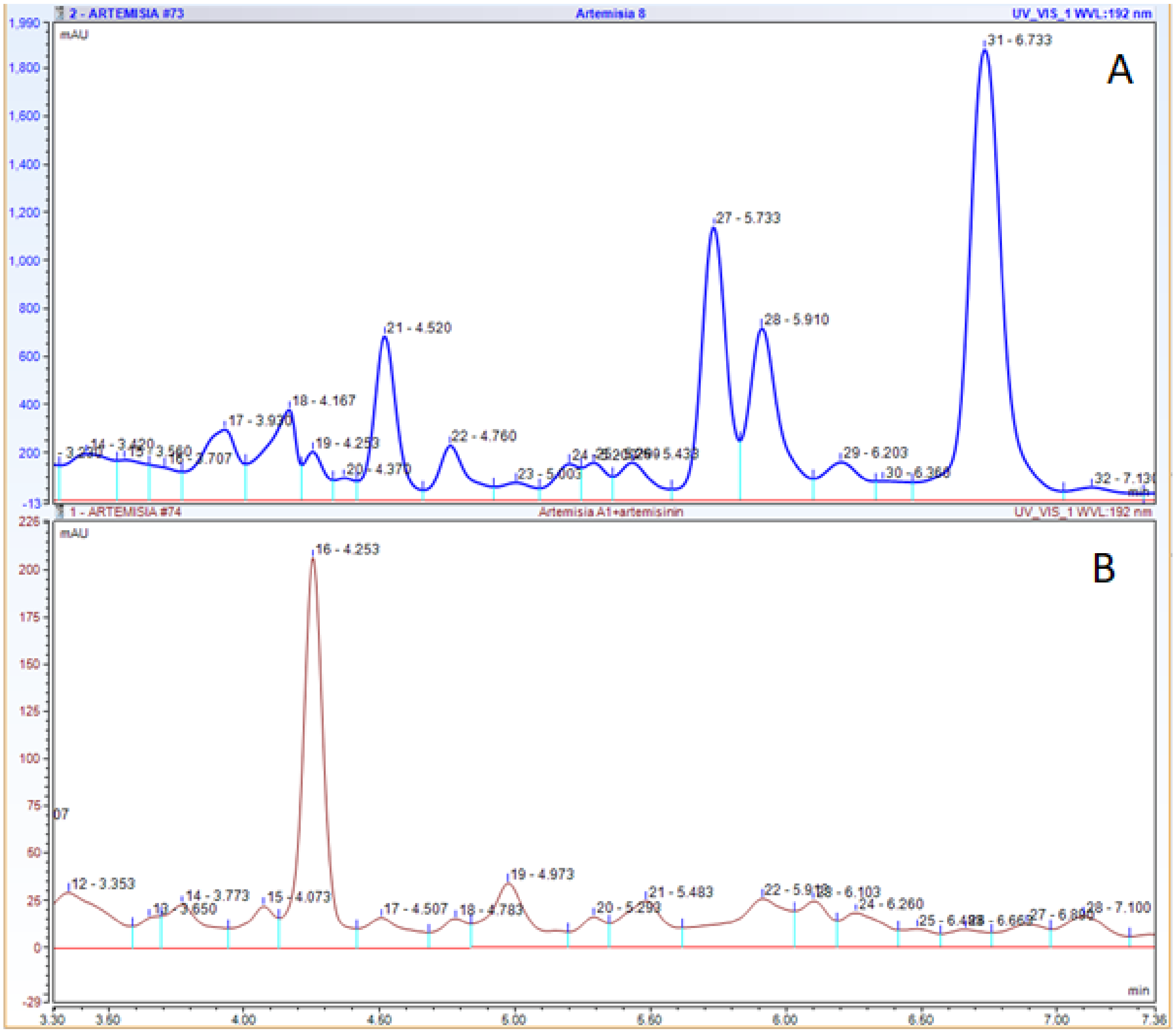

Figure S5. HPLC chromatogram of hexane extract of *Artemisia scoparia* (**A**) and mixture *Artemisia annua* and pure artemisinin (**B**).
